# 3D printing and medical imaging

**DOI:** 10.1002/jmrs.300

**Published:** 2018-09-02

**Authors:** Andrew Squelch

**Affiliations:** ^1^ Computational Image Analysis Group Curtin Institute for Computation Curtin University Perth Western Australia Australia

## Abstract

Three‐dimensional (3D) printing and medical imaging have a complementary association, the benefits and application areas of which are increasingly documented and further illustrated in this journal publication. Medical imaging data can be appropriately processed (i.e. segmented) to provide the geometric information from which accurate and realistic 3D medical models can be generated. The resulting models can be printed in a range of different materials to suit their use as phantoms in medical radiation and imaging studies, for medical imaging education and training or patient communication.

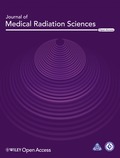

Three‐dimensional (3D) printing, which has been popularised in recent years with the advent of affordable desktop and personal 3D printers, originated 34 years ago as a form of rapid prototyping technology in the manufacturing industry.^1^ The primary use of the original photopolymer Stereolithography process being intended for the early phases of a production process, such as design proof of concept, scale model or production mould components. This initial application focus is still a key one today, but additional uses have since been proposed and identified including ones where the technique is used to generate the final item of production in its own right, for example an item of jewellery, a figurine, a dental crown and a mobile phone case.

3D printing technology is advancing rapidly and the capabilities and applications are being reported more widely.^2^, ^3^ These factors have led to the technology increasingly being applied in new areas and for new purposes. This situation is particularly true in the case of medical and healthcare areas where 3D printing is applied to producing:

- new and replacement personalised medical devices and implants;
- models for medical education and teaching;
- models for medical training and simulation;
- models for medical research; and
- models for pre‐operative planning.

There are other aspirational application areas, such as printing organ replacements with biological materials.^4^

The more advanced additive manufacture nature of the 3D printing process allows for the generation of model shapes that cannot be produced by any other manufacturing process, for instance compared to the earlier rapid‐prototype methods that relied on subtractive manufacture, for example carving or drilling in Computerised Numerical Control (CNC) machining.^5^ The additive nature lends itself to the organic nature of medical model shapes. The increasing variety of printing technologies, for example Stereolithography, Powder Bed Fusion, Fused Deposition Modelling,^2^ have gone hand‐in‐hand with providing a wide variety of material types, colours and physical properties. The technology has reached the stage of providing the capability to print multi‐material and multi‐component models. Several of these material types are now of great interest in the medical area, for example as direct anatomical replacement parts or as phantoms for pre‐operative planning, training and education purposes.

3D printing has found increasing association and engagement with medical imaging as evidenced in several publications in this issue of *Journal of Medical Radiation Sciences*. Initially as medical imaging, for example computed tomography (CT) and magnetic resonance imaging (MRI) scanning, provides an obvious source of the 3D geometry data from which medical models can be generated, in particular those required to be patient‐specific. Moreover, the quality, accuracy and viability of 3D models that are extracted from the medical image data for 3D printing are highly reliant on an optimal image segmentation process. Prompting investigations into medical image acquisition and processing workflows capable of generating 3D image volumes that dutifully comprise explicit features amenable to image segmentation. Finally for developing and evaluating improved radiation dosage and imaging contrast protocols whereby accurate 3D printed models are generated for use as phantoms in medical radiation and imaging studies.

The relevance and association of 3D printing with medical imaging is four‐fold:

- providing data to create realistic 3D medical models;
- establishing optimal scanning and image processing workflows for accurate image segmentation outcomes;
- use of 3D printed models as phantoms for medical radiation and imaging studies; and
- education and explaining the relationship and interpretation of anatomy from medical imaging.

The innovative use of 3D printing is demonstrated in the article by Abdullah et al.^6^ in which 3D printing was used to generate a reconfigurable heart insert phantom for cardiac CT protocols. This article illustrates two relationships of 3D printing with medical imaging, namely the use of medical imaging to obtain object‐specific source data for the generation of 3D models and the use of 3D models as phantoms for radiation and imaging protocols. The benefits of 3D printing are further demonstrated in the article by Lau and Sun systematically reviewing applications in the area of congenital heart disease, which provides examples of how 3D printing can be used effectively in pre‐operative planning, pre‐surgical simulation, medical education and communication in medical practice.^7^ Through analysis of 28 studies on 3D printing in congenital heart disease, this review article further confirms the accuracy of 3D printed models in replicating complex cardiac anatomy and pathology and the clinical value of using 3D printed heart models in managing patients with congenital heart disease.

The issue of model accuracy is raised and identified as an area requiring further study and, as previously noted, this aspect has reliance on and implications for the image acquisition process. 3D models destined for 3D printing must meet certain strict geometric criteria in order to be successfully printed. These are, essentially, that the model surface is ‘watertight’, that is it entirely encloses the solid part of the geometric shape without gaps, and that minimum wall thicknesses are honoured.

The benefits and potential use of 3D printing in the area of medical imaging are increasingly documented and further illustrated in this journal publication. The material selected for the 3D printed model will be dependent on the intended outcome and if realism of touch or imaging contrast is required. Some materials permit printing of models with outer transparency for convenience of viewing internal structures printed in different colours suitable for pre‐operative planning and medical education purposes.^8^ Other materials provide printed models that have a rubbery tissue‐like characteristic suitable for pre‐operative planning, surgical simulation and patient communication purposes.^7^

Nonetheless the costs of 3D printing should be borne in mind as noted in the review of 3D printing in the area of congenital heart disease, as costs can range from a few tens of dollars to several hundred dollars and in some cases even thousands of dollars. The relatively high cost encountered in one study resulted in opting for a scaled‐down model and associated loss of life‐like illustration of the cardiac anatomy. The choice of material, as well as the size and associated volume of material, affects the cost to print a given 3D model, as well as the ultimate quality, realism and fidelity of the output model.

As evidenced by the articles published in this journal and elsewhere, 3D printing and medical imaging have a complementary and meaningful association that will likely still see many technical contributions and advances being made.

## Conflict of Interest

The author declares no conflict of interest to declare.
